# Frequent torsades de pointes in a child with novel *AKAP9* mutation: A case report and literature review

**DOI:** 10.3389/fped.2022.1027177

**Published:** 2023-01-09

**Authors:** Yefeng Wang, Chao Zuo, Xiang Wang, Yunbin Xiao, Qiming Liu, Zhi Chen

**Affiliations:** ^1^ Department of Cardiology, Hunan Children’s Hospital, Changsha, China; ^2^ Department of Cardiology, Second Xiangya Hospital, Central South University, Changsha, China

**Keywords:** torsades de pointes (TdP), long QT syndrome (LQTS), *AKAP9* gene, case report, implantable cardioverter defibrillator (ICD)

## Abstract

**Introduction:**

The aim of the present study is to report the diagnosis and treatment of a rare case of frequent torsades de pointes (Tdp) in a child with a novel *AKAP9* mutation. A 13-year-old girl suffered from repeated syncope and frequent Tdp. An electrocardiogram (ECG) showed frequent multisource premature ventricular contractions with the R-ON-T phenomenon. The QTc ranged from 410 to 468 ms. The genetic test indicated a heterozygous mutation, namely, c.11714T > C (p.M3905T), in the *AKAP9* gene, which is a controversial gene in long QT syndrome. After treatment with propranolol, recurrent syncope occurred, and the patient received an implantable cardioverter defibrillator (ICD). Due to frequent electrical storms at home, the child was additionally treated with propafenone to prevent arrhythmia. The antitachycardia pacing (ATP) function in the ICD was turned off, and the threshold of ventricular tachycardia (VT) assessment was adjusted from 180 beats/min to 200 beats/min. The patient was followed up for 12 months without malignant arrhythmia and electric shock.

**Conclusion:**

Genetic testing may be a useful tool to determine the origin of channelopathy, but the results should be interpreted in combination with the actual situation. Rational parameter settings for the ICD and application of antiarrhythmic drugs can reduce the mortality rates of children.

## Introduction

Torsades de pointes (Tdp) is a life-threatening ventricular tachyarrhythmia characterized by a continuously changing QRS complex morphology, with the electrical axis twisting around the isoelectric line. Tdp is associated with a prolonged QT interval and may be preceded by T-wave alternans (1). Long QT syndrome (LQTS) is the most common hereditary ion channel disease in childhood, and it is characterized by Tdp, syncope, and sudden death. There are currently 17 genes known to cause LQTS, and their clinical manifestations are different (2). The present report discusses the diagnosis and treatment of a rare case of LQTS with a novel *AKAP9* mutation in a child, and it summarizes the relevant experience and literature analysis. The parents of the child signed an informed consent, and this study was approved by the Medical Ethics Committee of the hospital (Hunan Children's Hospital, Changsha, China).

## Case presentation

A 13-year-old girl was admitted to the hospital because of dizziness for 3 days and syncope twice. She experienced syncope first during school recess, which lasted for approximately 10 min, accompanied by salivation and urinary incontinence. She was immediately sent to the emergency department of a local hospital. During hospitalization, she suffered syncope for a second time, and she was then transported to our hospital by ambulance.

**Figure 1:**
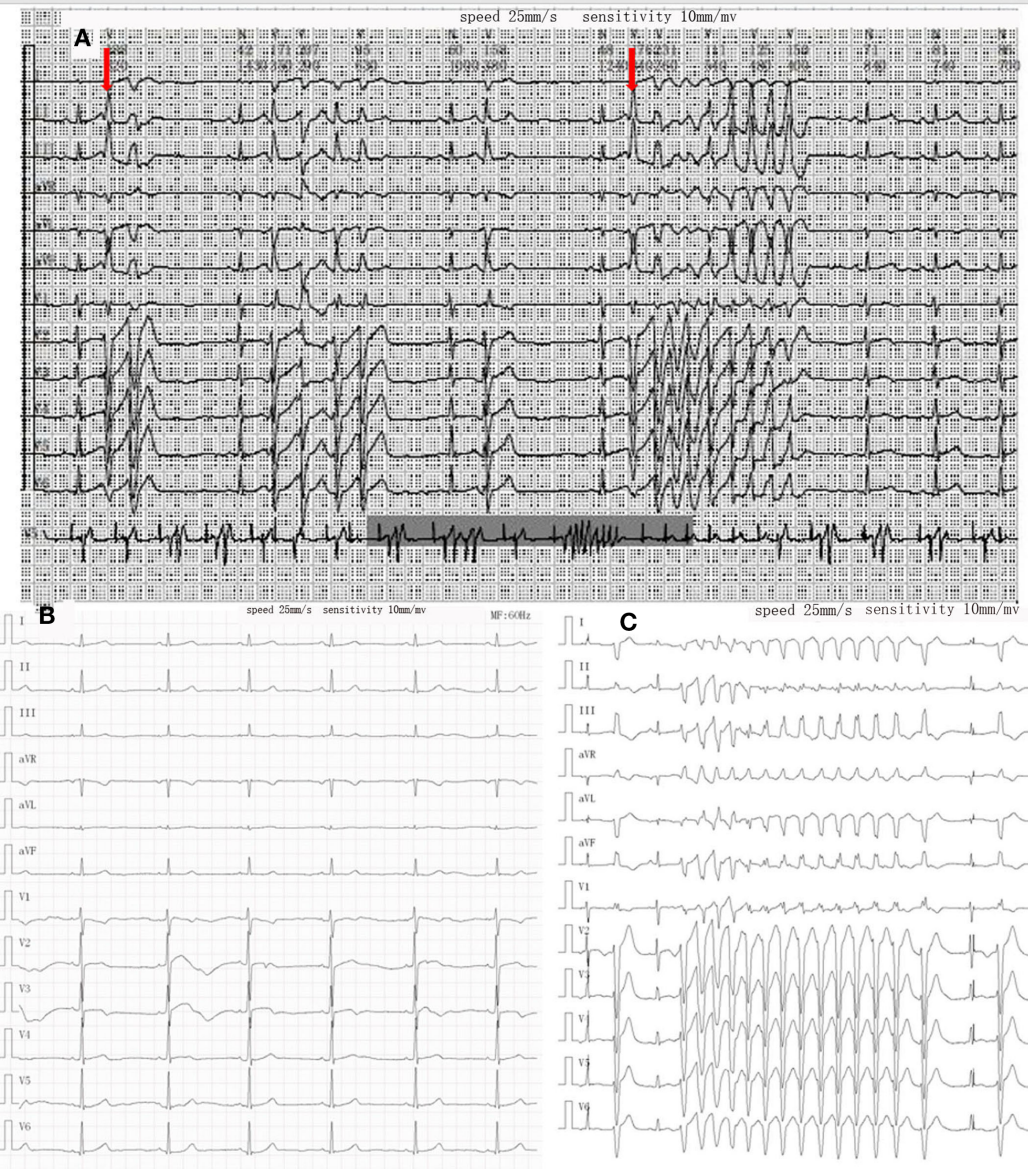
Changes in ECG during treatment. (**A**) Holter on admission showed R on T phenomenon (red arrow) and torsades de pointes. (**B**) After treatment of propranolol and magnesium sulfate, the QT interval was in the normal range, and no obvious ventricular premature beats were found. (**C**) Recurrence of torsades de pointes episode during oral propranolol.

**Figure 2:**
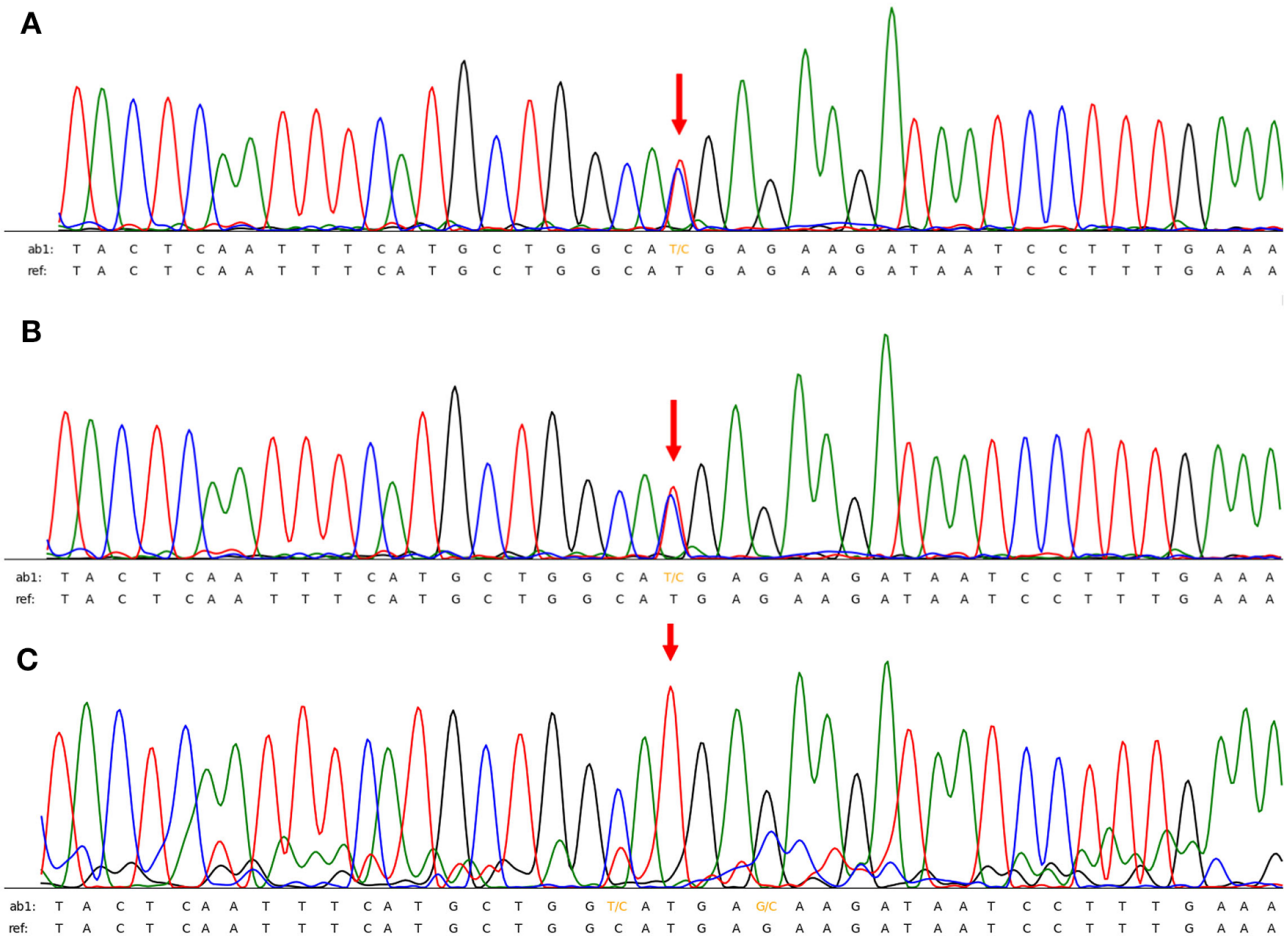
(**A,B**) the patient and her mother had a heterozygous mutation of *AKAP9* gene mutation of c.11714T > C (p.M3905T). (**C**) Her father had no relevant genetic mutations.

After admission, physical examination revealed that the child was conscious with a normal heart rate but an irregular heartbeat. Echocardiography showed normal left ventricular systolic function and a normal cardiac structure. Multiple bedside electrocardiograms (ECGs) showed frequent multisource premature ventricular contractions. Holter ECG showed frequent multisource premature ventricular contractions (43,729 beats/day, with some occurring in pairs and some occurring in doublet or triplet patterns) with the R-ON-T phenomenon and Tdp (Figure 1A). The QTc ranged from 410 to 468 ms. For treatment, the patient was administered an intravenous infusion of magnesium sulfate (0.5–1.0 mg/kg h) and potassium to maintain serum potassium concentration at 4.5–5 mmol/L. She received oral propranolol tablets (0.5 mg/kg) every 8 h. After 1 week of treatment, the child recovered and did not experience palpitations or dizziness in the hospital. An ECG showed that the premature ventricular contractions were significantly reduced and that no ventricular tachycardia (VT) occurred, and the QTc was 416 ms (Figure 1B). Occasionally, a biphasic T wave was observed in Lead V2 in this patient in sinus rhythm. Second-generation gene sequencing was performed after receiving approval by the Medical Ethics Committee and parental informed consent. As a result, a heterozygous mutation, namely, c.11714T > C (p.M3905T), in the *AKAP9* gene of the patient was detected, which was inherited from her mother (Figure 2), whose ECG was normal without QT prolongation and no history of syncope. This variant has not been previously described in the literature and has also not been previously reported in the Human Gene Mutation Database (HGMD). No other possible causative mutations were found, and no history of related genetic diseases was detected in the family.

**Figure 3:**
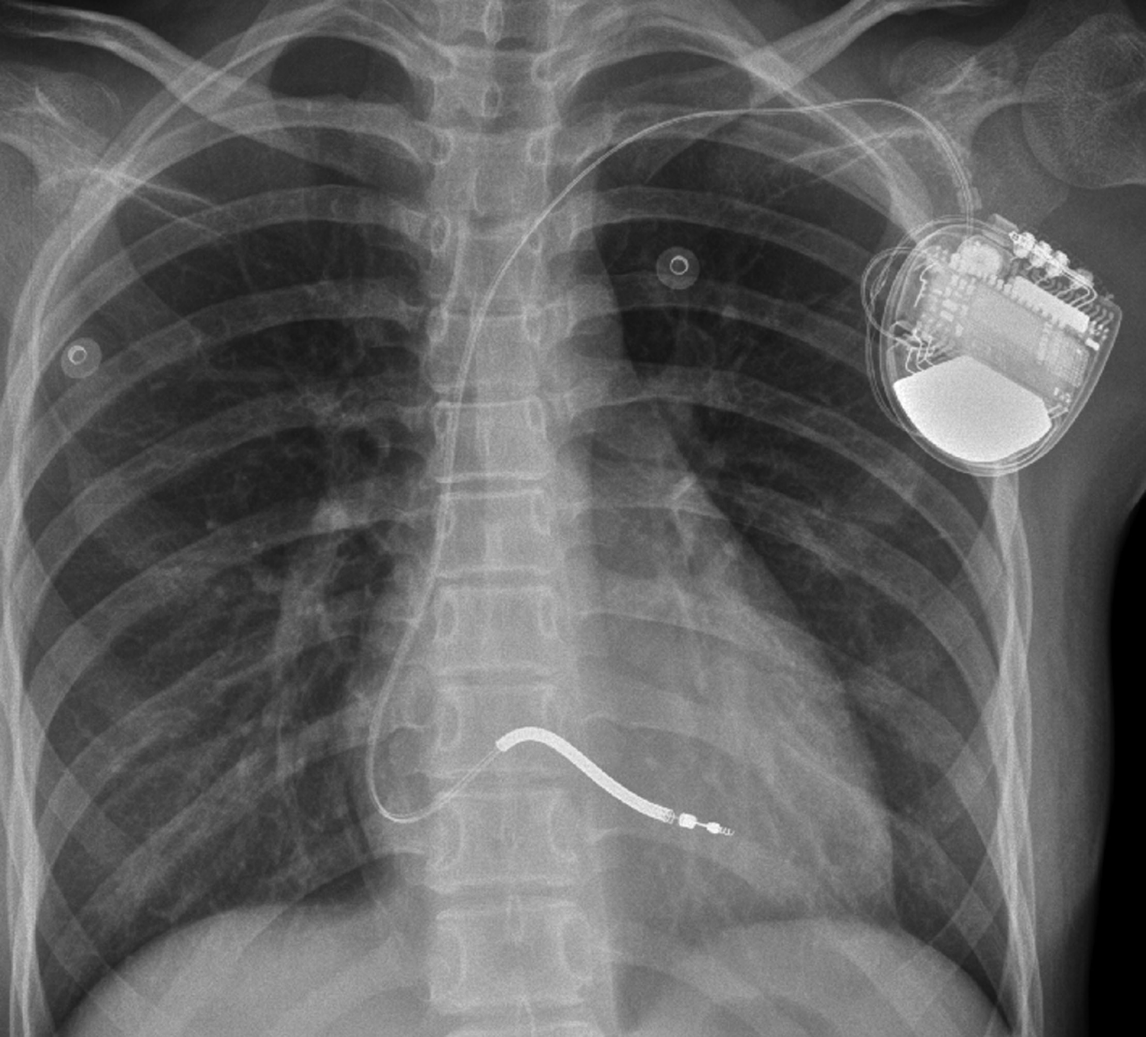
Chest x-ray after the implantation of the implantable cardioverter-defibrillator.

After 9 months, the child suffered from syncope and convulsions again during housework. She was transferred to our hospital after cardiopulmonary resuscitation for 2 h. The ECG showed frequent multisource VT with Tdp (Figure 1C). Temporary subclavian pacing was given to the patient, and vasoactive drugs were used to maintain circulatory stability. Oral propranolol tablets were adjusted to 1 mg/kg every 8 h, and the patient received intravenous potassium and magnesium supplementation. The Holter ECG showed that the incidence of ventricular arrhythmia was 19,924 beats in 24 h with no occurrence of Tdp. Considering that the child had syncope after drug treatment, she was implanted with an implantable cardioverter defibrillator (ICD) under general anesthesia after 7 days of stabilization (Figure 3). Her parents were informed that she needed activity restriction and exercise reduction.

Two weeks after discharge from the hospital, the patient developed obvious palpitations, fatigue, dizziness, and a sense of electric shock at night. Because she experienced several suspicious electric shock events, she was readmitted to our hospital. The bedside program control of the ICD confirmed that the child had multiple VT at home, and electrical cardioversion terminated the tachycardia after antitachycardia pacing (ATP) failed. In order to alleviate her anxiety, the ATP function was turned off, the VT judgment threshold was adjusted from 180 beats/min to 200 beats/min, and the energy of the first electric shock was lowered from 30 to 20 J. To reduce the incidence of ventricular arrhythmias, the patient was prescribed oral propafenone tablets (3 mg/kg) every 8 h. A repeated Holter ECG showed that the frequency of ventricular arrhythmias (3,387 beats/day) was significantly lower than before and that the ventricular pacing function was normal. The morphology of the T wave was normal in the lateral precordial leads. No malignant arrhythmia and electric shock events occurred during the 1-, 3-, and 6-month follow-ups. The child returned to school with restricted activities, and her parents were satisfied with the treatment provided.

## Discussion

**Figure 4:**
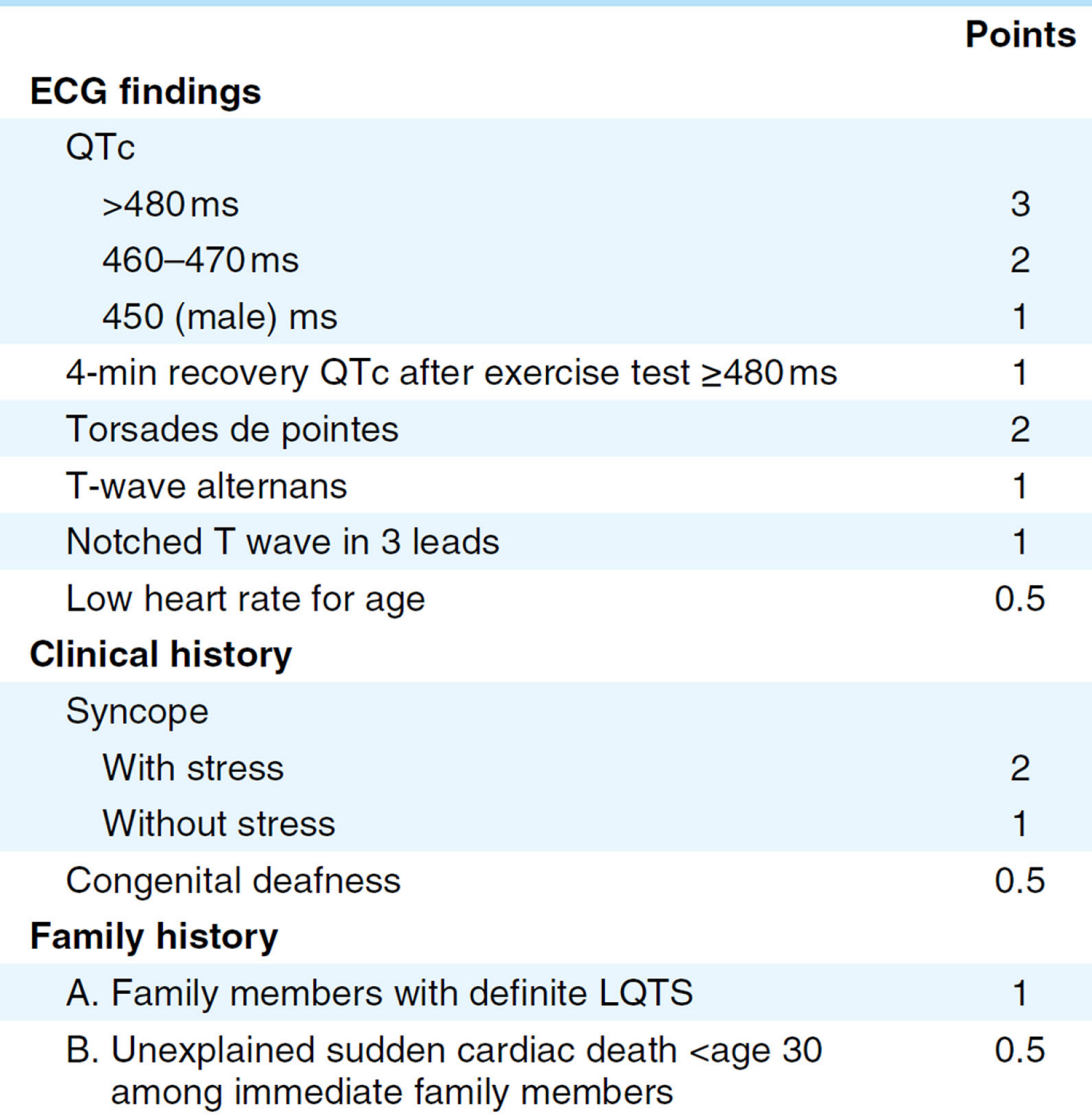
Diagnostic criteria for long QT syndrome (LQTS) (the Schwartz score). Definite LQTS is defined by an LQTS score of ≥3.5 points.

The diagnosis of LQTS is mainly based on electrocardiographic manifestations, clinical manifestations, and family history. A Schwartz score of ≥3.5 is considered for the diagnosis of LQTS (Figure 4) (2). In the present case, LQTS was diagnosed based on QT interval prolongation, history of Tdp, and syncope with stress. Based on the Schwartz scoring standard, the patient received a score of 6 points and met the diagnostic criteria of LQTS. Ion channel gene detection also aids in genotyping and guiding the treatment of LQTS (4). In the present case, we identified a heterozygous mutation, namely, c.11714T > C (p.M3905T), in *AKAP9*, which has not been reported in previous cases.

Previous studies have named LQTS caused by *AKAP9* gene mutation as LQTS type 11. However, Adler et al. (5) analyzed a large sample and reported that among the 17 known genes that cause LQTS, only 3, namely, *KCNQ1*, *KCNH2*, and *SCN5A*, are clearly related to the occurrence of LQTS. Thus, it remains controversial whether *AKAP9* can cause LQTS. Previous studies on the *AKAP9* gene utilized a candidate gene approach compared with an unbiased genome-wide methodology used in this study. Recent studies have suggested that the protein encoded by the *AKAP9* gene is protein kinase A-anchored protein 9 (Yotiao protein) and that its function is mainly to act as a scaffold protein necessary for the assembly of several protein kinases and phosphatases on the centrosome and Golgi apparatus (6). The Yotiao protein forms a macromolecular complex with the voltage-gated potassium channel alpha subunit, Kv7.1 (also known as *KCNQ1*), and its associated beta subunit, KCNE1, which is responsible for the slow activation of delayed rectifier K+ currents and is a modifier of the clinical phenotype of LQTS (7, 8). The *AKAP9* gene not only alters QTc duration but also affects the risk and severity of cardiac events. In this case, the patient presented with frequent Tdp and multisource VT without significant QTc prolongation, which also indicated that the ECG manifestation of LQTS 11 was atypical but had a high degree of malignancy. However, the child's mother, who harbors the same heterozygous mutation in the *AKAP9* gene, had no LQTS-related symptoms, which suggested that *AKAP9*-associated LQTS is debatable, warranting further research to determine whether there are other unknown genes and influencing factors.

Treatments for LQTS include medication, implantable device therapy, and cardiac sympathetic denervation. Beta-blockers have been widely used as first-line drug therapy for congenital LQTS, and the mechanism of their antiarrhythmic effect is to reduce or prevent the increase in cardiac transmural repolarization dispersion that occurs during intense sympathetic stimulation. However, if serious cardiovascular events still occur after an adequate dose of propranolol, an ICD or cardiac sympathetic denervation should be considered (3). Postoperative electrical storm is a common complication after ICD implantation in children, but the incidence in children is unknown. In adults, the incidence of electrical storm after ICD can be as high as 10%–60% (9), and it is mainly related to myocardial ischemia, electrolyte disturbance, sympathetic nerve excitation, and drugs (10). In this case, the child suffered from ICD electric shock during hyperbaric oxygen therapy as well as repeated electrical storms after painful stimulation and tension, which may have been related to sympathetic nerve excitation and the failure of beta-blockers to effectively control arrhythmia. Propafenone is a class Ic antiarrhythmic drug with strong membrane stability, competitive beta-receptor blockade, and calcium channel blockade, and it quickly interferes with sodium channels (11). In this case, the incidence of ventricular arrhythmias was significantly reduced after the addition of propafenone. However, attention should be paid to the children's QT interval and cardiac function to avoid cardiac insufficiency and prolongation of the QT interval when propafenone is combined with propranolol (12).

Currently, there is a lack of high-evidence guidelines or consensus on parameter settings for ICDs in children with cardiac channelopathies. For children with ICD implantation, especially when the diagnosis of cardiac channelopathies is clear and malignant arrhythmias have occurred in the past, the threshold for VT assessment in the ICD should not be lower than 188 beats/min or higher than the recorded VT (13). In this case, the VT threshold of the ICD was adjusted from 180 beats/min to 200 beats/min to avoid frequent electric shocks. The ATP function is recommended to reduce ICD discharge after ICD implantation in adults. However, in cardiac channelopathies, Tdp or ventricular fibrillation (VF) is the main attack and not monomorphic VT. Thus, in children with ATP and obvious symptoms of palpitations and anxiety, malignant arrhythmias are more like to occur. In the present case, after ATP function of the ICD was turned off, no electrical storm occurred again.

In conclusion, the diagnosis of LQTS in children needs to be combined with ECGs, clinical manifestations, and family history. Early genetic examination is helpful for typing and guiding treatment, but the genetic results need to be interpreted in combination with the actual situation. ICD implantation, as a means of preventing sudden death of children with cardiac syncope, can significantly reduce the sudden death rate. Rational parameter settings and application of antiarrhythmic drugs can reduce the mortality rates of children.

## Data Availability

The original contributions presented in the study are included in the article/supplementary material; further inquiries can be directed to the corresponding author.
